# P-297. Prospective Study on Microbiological Profile of Nosocomial Infections due to Gram negative lactose non-fermentative Bacteria in a Tertiary Care Hospital in South India

**DOI:** 10.1093/ofid/ofae631.500

**Published:** 2025-01-29

**Authors:** Sriram Radhakrishnan, Vaishnavi Velmani, K Shailaja, Sureshkumar Dorairajan

**Affiliations:** C.L.Baid Metha College of Pharmacy, Affiliated to the Tamil Nadu Dr. MGR Medical University, Chennai, Tamil Nadu, India; C.L.Baid Metha College of Pharmacy, Affiliated to the Tamil Nadu Dr. MGR Medical University, Chennai, Tamil Nadu, India; C.L.Baid Metha College of Pharmacy, Affiliated to the Tamil Nadu Dr. MGR Medical University, Chennai, Tamil Nadu, India; Best of IDs, Chennai, Tamil Nadu, India

## Abstract

**Background:**

Non-fermentative gram-negative bacteria (NFGNB) infections that are acquired in hospitals have particularly concerning characteristics; identifying these features can aid in prevention and improve the standard of care provided within hospital premises. This study aims to find out the nosocomial infections caused by NFGNB, its sensitivity pattern, length of culture reporting time, and patients with ICU stay.

**Figure:**
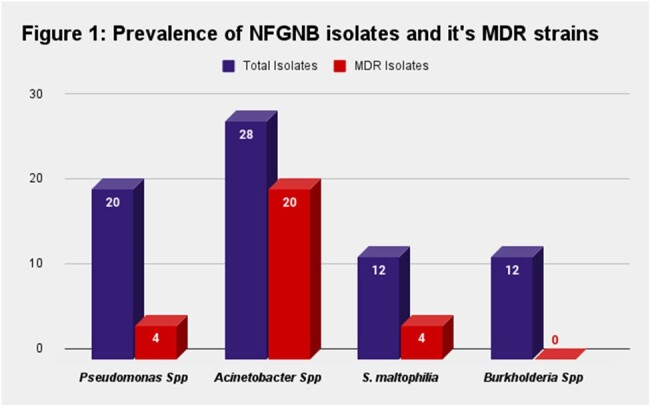

**Methods:**

A prospective observational study was conducted in a tertiary care hospital located in southern India during the 4 months of January-April 2024. Data were taken from the Department of Microbiology regarding the prevalence of NFGNB isolates from different culture samples and their sensitivity pattern. The number of MDR isolates throughout the four months, their susceptibility pattern, and the duration of culture reporting time for all NFGNB isolates were examined. Descriptive analysis was used to look at patient demographics, inpatient department, and ICU admission. Statistical analysis was done by Microsoft Excel.

**Figure:**
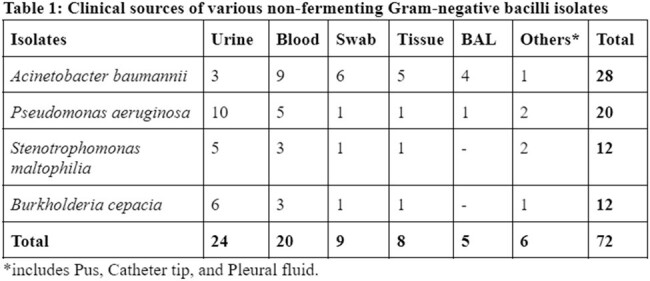

**Results:**

Total 72(18.6%) samples were recorded as NFGNB from 386 culture positive samples isolated from the Department of Microbiology. The most common isolate was *Acinetobacter spp* (38.8%) followed by *Pseudomonas spp* (27.7%). The mean of our study participants was found to be 55 years, with a male: female ratio 2:1. The General Medicine department showed maximum isolates i.e, 25% followed by Hepatology department i.e, 16.6%. Maximum NFGNB isolates are from Urine sample (33.3%) followed by Blood sample (27.8%). The mean time of culture reporting for NFGNB was 72 hours. Out of 72 NFGNB samples, 28(38.8%) were Multi-drug resistant isolates and 29(40.2%) required ICU admission.

**Figure:**
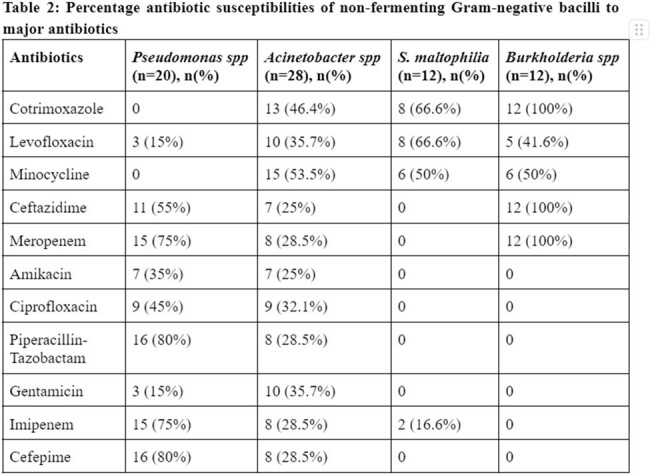

**Conclusion:**

NFGNB function as nosocomial pathogens, their startling resistance is a concern to epidemiology and medical care. These isolated pathogens have been manually reported for a considerable amount of time. Rapid culture identification methods would assist identify resistant diseases early and improve patient outcomes in low- and middle-income countries. Future studies should focus on implementation of approaches to reduce the incidence and resistance of nosocomial infections in LMICs.

**Disclosures:**

**All Authors**: No reported disclosures

